# The M3 Phosphorylation Site Is Required for Trafficking and Biological Roles of PIN-FORMED1, 2, and 7 in *Arabidopsis*

**DOI:** 10.3389/fpls.2016.01479

**Published:** 2016-09-28

**Authors:** Daeeun Ki, Daisuke Sasayama, Hyung-Taeg Cho

**Affiliations:** Cell Differentiation Lab, Department of Biological Sciences, Seoul National UniversitySeoul, Korea

**Keywords:** auxin, auxin transport, auxin transporter, PIN-FORMED (PIN), protein phosphorylation, protein trafficking

## Abstract

Asymmetrically localized PIN-FORMED (PIN) auxin efflux carriers play key roles in regulating directional intercellular auxin movement, generating local auxin gradients, and diverse auxin-mediated growth and development. The polar localization of PINs is controlled by phosphorylation in the central hydrophilic loop (HL) of PINs. Although the M3 phosphorylation site, including phosphorylatable 5 Ser/Thr residues, is conserved among long HL-PINs, its native role has only been characterized in PIN3. In this study, we examined the role of M3 phosphorylation site of PIN1, PIN2, and PIN7 in intracellular trafficking, phosphorylation, and biological functions of those PINs in their native expressing tissues. Phosphorylation-defective mutations of the phosphorylatable residues in the M3 site of PIN1-HL led to alteration in subcellular polarity of PIN1 and caused defects in PIN1-mediated biological functions such as cotyledon development, phyllotaxy of vegetative leaves, and development of reproductive organs. The M3 mutations of PIN7 interfered with its polar recycling in the root columella cell in response to gravity stimulus and partially disrupted root gravitropism. On the other hand, the M3 site of PIN2 was shown to be necessary for its targeting to the plasma membrane. *In vitro* phosphorylation assay showed that the M3 phosphorylation residues of PIN1 are the partial targets by PINOID kinase. Our data suggest that the M3 phosphorylation site is functionally conserved among long HL-PINs by playing roles for their subcellular trafficking and auxin-mediated developmental processes.

## Introduction

Auxin is the key hormone for diverse developmental processes in plants. Differential distribution of auxin within plant tissues is essential for auxin-mediated development (Grunewald and Friml, 2010; Ganguly et al., 2012b). Local auxin gradients can be formed by auxin transporter proteins such as PIN-FORMED (PIN) that are asymmetrically localized at the plasma membrane (PM) (Wiśniewska et al., 2006). The *Arabidopsis* genome encodes eight PIN proteins that can be classified into two subgroups by the size of central hydrophilic loop (HL) domain. PIN1, PIN2, PIN3, PIN4, PIN6, and PIN7 have a long HL (298–377 residues; long PINs), whereas PIN5 and PIN8 have a relatively short HL (27–46 residues; short PINs) (Křeček et al., 2009; Ganguly et al., 2012b). While short PINs localize to either internal compartments or PM depending on molecule and cell type, long PINs predominantly localize to the PM with polarity (Ganguly et al., 2010, 2014).

Developmental and environmental cues affect the subcellular polarity of PINs (Friml et al., 2002b, 2003; Benková et al., 2003). Long PINs show different subcellular polarity depending on cell type and PIN species (Wiśniewska et al., 2006; Ganguly et al., 2014). PIN1, PIN3, PIN4, and PIN7 typically localize at the basal PM toward the root tip in the *Arabidopsis* root stele and meristem region (Friml et al., 2002a,b; Blilou et al., 2005). In contrast, PIN2 localizes apically (toward the shoot) in root epidermis, elongating cortex, and lateral root cap cells and basally (toward the root) in meristematic cortex cells (Müller et al., 1998). In root columella cells where PIN3 and PIN7 are expressed, PIN3 and PIN7 dynamically change their polarity in response to gravity stimulation (Friml et al., 2002b; Kleine-Vehn et al., 2010).

The subcellular polarity of PINs is determined by polar trafficking of PIN proteins (Dhonukshe et al., 2008). The HL domain of long PINs has been a target as the molecular cue for regulation of intracellular trafficking and thus subcellular polarity of PIN proteins. In particular, phosphorylation/dephosphorylation of PIN-HL plays a decisive role in PINs’ trafficking and biological functions (Ganguly et al., 2012b). AGC protein kinase family members have been implicated in phosphorylation of PIN efflux carriers and ABC transporters (Zhang and McCormick, 2008; Ganguly et al., 2012b; Barbosa and Schwechheimer, 2014). Coupled phosphorylation/dephosphorylation by PINOID (PID) (and its closely related AGCVIII kinases) and protein phosphatase 2A (PP2A), respectively, has been well studied for PIN polar targeting (Friml et al., 2004; Lee and Cho, 2006; Michniewicz et al., 2007).

Several phosphorylation sites in the PIN-HL have been functionally characterized (Michniewicz et al., 2007; Dhonukshe et al., 2010; Huang et al., 2010; Ganguly et al., 2012a; Zourelidou et al., 2014). Our previous study with PIN3 showed that the M3 phosphorylation site of the HL domain is required for PID-mediated phosphorylation, subcellular polarity, and biological function of PIN3 (Ganguly et al., 2012a). The M3 phosphorylation site of PIN3 includes 5 phosphorylatable Ser/Thr residues over the 18 residue-long region of PIN3-HL. Although the phosphorylation residues are mostly conserved, this M3 region shows some variations among different PINs, where a Ser residue is missing in PIN2 and the Ser/Thr-flanking sequences vary in different PIN molecules (Supplementary Figure S1). The M3 phosphorylation site affected the trafficking of PIN1, PIN2, and PIN7 when they were ectopically expressed in the root hair cell (Sasayama et al., 2013). However, it has still remained to show whether the M3 phosphorylation site generally influences the biological functions of long PINs. In order to answer this question, we have complemented the loss-of-function mutants of *pin1*, *pin2*, and *pin7* with wild-type (WT) or M3-mutant versions of those PINs and analyzed the mutation effects on each PIN’s own biological function.

## Materials and Methods

### Plant Materials and Growth Conditions

*Arabidopsis thaliana* (Columbia ecotype) was used as the WT plant in this study. *Arabidopsis* plants were transformed using *Agrobacterium tumefaciens* strain C58C1 (pMP90) (Bechtold and Pelletier, 1998). All seeds were vernalized at 4°C for 3 days and germinated at 22°C under a 16-h-light/8-h-dark photoperiod. Seeds were grown on the medium containing 4.3 g/L Murashige and Skoog (MS) nutrient mix (Sigma–Aldrich), 1% sucrose, 0.5 g/L MES (pH 5.7 with KOH), and 0.8% agarose. Transformed plants were selected on the hygromycin-containing (30 μg/mL) medium.

### Transgene Constructs

The binary vector pCAMBIA 1300-NOS with modified cloning sites (Lee et al., 2010) was used for transgene construction. *ProPINs:PINs:GFP*, *ProPINs:M3PINs:GFP*, and *ProPINs:3m1PINs:GFP* constructs were generated by replacing the *ProE7* fragment of *ProE7:PINs:GFP*, *ProE7:M3PINs:GFP*, and *ProE7:3m1PINs:GFP* (Sasayama et al., 2013) with the promoter region of *PIN*s (*ProPIN*s). *ProPIN*s were amplified by PCR using the *Arabidopsis* genomic DNA as template and the primers listed in Supplementary Table S1.

For heterologous expression of PIN-HL proteins in *Escherichia coli* (*E. coli*) for the *in vitro* phosphorylation assay, the *PIN-HL* part was obtained from *Arabidopsis* cDNA by using the primers in Supplementary Table S1 and cloned into the *pGEX-4T-1* vector (GE Healthcare, Inchon, Korea) for fusion with glutathione *S*-transferase (GST) at the N-terminus. The protein kinase PID used in the kinase assay was as described in Ganguly et al. (2012a).

All constructs were confirmed by nucleotide sequencing, and at least five independent transgenic lines for each construct were analyzed.

### Observation of Biological Parameters

Phyllotatic analysis was conducted by numbering the 3-week-old vegetative leaves from early to late developmental stages, and the angles between leaves were measured by the Leica Application suite (v.2.8.1). The seed number per silique was measured under the Leica MZFLIII dissecting microscope. For the root gravitropism assay, 3-day-old seedlings grown vertically in the light were turned 90° for the indicated period of time (2–10 h) in the dark condition before observing root bending.

### Confocal Microscopy of Fluorescent Proteins

Observation of fluorescent reporters was conducted as described previously (Ganguly et al., 2010; Ganguly et al., 2012a). To observe cytological effects of brefeldin A (BFA), seedlings were pretreated with cycloheximide (50 μM) for 30 min and followed by BFA (25 μM) treatment for 30 min. BFA was dissolved in DMSO (0.05% final), and control treatments included 0.05% DMSO. Quantification of PM-localized *PIN:GFP* signals was performed using the histogram function of Adobe Photoshop CS6 (Adobe Systems).

To observe gravitropic changes of PIN7:GFP localization in root columella cells, 4-day-old seedlings were transferred to a slide glass containing half-strength MS medium and grown for 24 h vertically in the dark condition. Gravity stimulation was applied for the indicated time period by turning the slide 90°, subsequently the changes of PIN7:GFP signal were analyzed under a confocal microscope. To visualize cell boundaries, FM4-64 (2 μM) was applied for 3 min.

### *In vitro* Phosphorylation Assay

Expression of GST-fused kinase (PID) and substrates (WT and M3-mutated PIN1-HL) in *E. coli* (BL21DE3) cells was induced by 1 mM isopropyl β-D-1-thiogalactopyranoside for 3 h at 28°C, and the cells were harvested by centrifugation at 3900 *g* for 10 min. The harvested cells were lysed with B-PER bacterial protein extraction reagent (Thermo Scientific, Seoul, Korea) with the Halt protease inhibitor complex (Santa Cruz), and the GST-fused proteins were purified using the GST-bound agarose resin (Elpis biotech).

For *in vitro* phosphorylation assays, l μg of kinase and substrate proteins, respectively, were mixed in 30 μl kinase buffer (25 mM Tris–HCl pH 7.5, 1 mM DTT, 5 mM MgCl_2_, 100 uM sodium orthovanadate, 30 mM β-glycerophosphate) and 1 μCi [γ-32P] ATP for 1 h at 30°C and, the reaction was halted by adding 5 μl of SDS-PAGE loading buffer and boiling for 10 min. The reaction samples were then separated in 10% acrylamide gels and stained with Coomassie Brilliant Blue. Autoradiography was performed using BAS imaging plate (BAS 2040, Fujifilm) and Bio-imaging Analyzer (BAS-2500, Fujifilm). The intensity of the phosphorylation bands was estimated by ImageJ software^1^ (the National Institutes of Health, Bethesda, MD, USA). To estimate the phosphorylated band intensity, the background intensity value of the same lane was subtracted from the intensity value of the phosphorylated PIN band.

## Results and Discussion

Among 5 Ser/Thr residues of the M3 site of PIN3, single or double mutations of them barely showed the mutation effect on PIN3’s trafficking and biological roles, indicating their redundant function (Ganguly et al., 2012a). However, the mutation of the first 3 Ser residues (so called 3m1; Supplementary Figure S1) considerably influenced PIN3’s trafficking and biological role, though the effect of whole 5 mutations (M3) was greater than that of 3m1 (Ganguly et al., 2012a). In this study, to know the role of M3 phosphorylation residues in other long PINs, M3 and 3m1 mutations of PIN1, PIN2, and PIN7 were generated and compared with the WT PINs for their subcellular polarity and biological functions.

### Proper PIN1 Trafficking and Polarity Require the M3 Phosphorylation Site

In the root, PIN1 is mainly localized at the basal PM of vascular cells and the basal and inner lateral sides of pericycle and endodermal cells (Gälweiler et al., 1998). In order to study the effect of M3 and 3m1 phosphorylation sites on subcellular localization of PIN1, WT PIN1 and mutated PIN1 at the M3 or 3m1 region (M3PIN1 or 3m1PIN1; Supplementary Figure S1) of the HL domain were expressed under its own promoter (*ProPIN1*) in the *pin1* mutant background.

**FIGURE 1:**
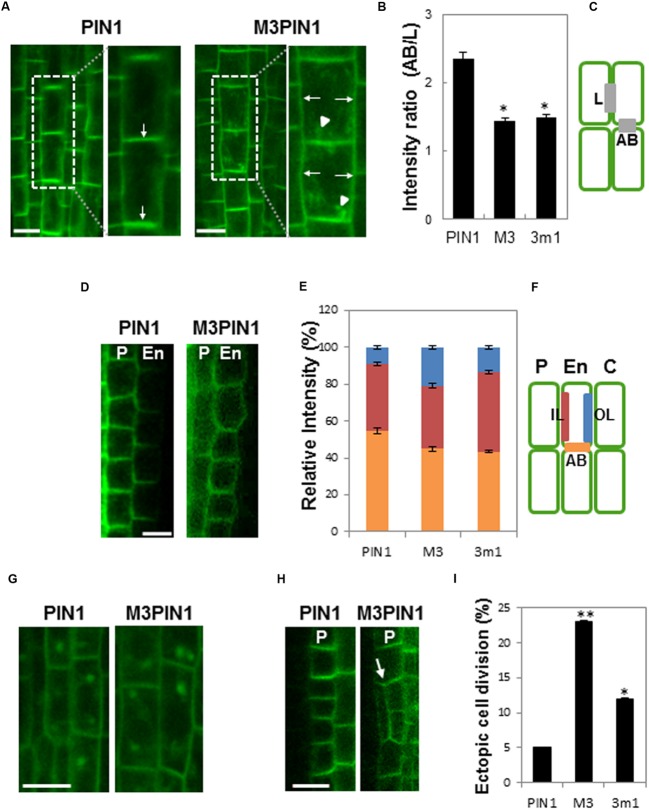
**Effects of M3 or 3m1 mutation on the polarity and trafficking of PIN1. (A)** Representative confocal images of PIN1 (*ProPIN1:PIN1:GFP*) and M3PIN1 (*ProPIN1:M3PIN1:GFP*) in root vascular cells. Arrows indicate basally intensified and laterally depolarized localization of PIN1 and M3PIN1, respectively. Arrowheads indicate internalized compartments of M3PIN1. Bar = 10 μm. **(B,C)** Quantification of the PIN polarity shown in **(A)**. The fluorescence ratio of apical and basal (AB)/lateral (L) membranes sides (gray bars in **C**) of PIN1, M3PIN1, and 3m1PIN1 (*ProPIN1:3m1PIN1:GFP*). Data represent means ± SE (*n* = 24–27 cells from 7 to 8 roots for each construct). Asterisks indicate that differences are significant from the PIN1 value (*P* < 0.0001). **(D)** Representative confocal images showing the subcellular localization of PIN1 and M3PIN1 in pericycle (P) and endodermis (En) cells of the root. Bar = 10 μm. **(E,F)** Quantification of the PIN polarity in the endodermis shown in **(D)**. The fluorescence ratio of outer lateral (OL in blue), inner lateral (IL in red), and apical and basal (AB in orange) membrane as shown in **(C)** was estimated with wild-type (WT) and mutant PIN1 lines (C, cortex). Data represent means ± SE (*n* = 25 cells from 8 to 9 roots for each construct). **(G)** Representative confocal images showing BFA compartments of PIN1- and M3PIN1 in root vasculature cells. Bar = 10 μm. **(H)** Representative confocal images of ectopic cell division in the WT or mutant PIN1-expressing root pericycle (P). Arrow indicates ectopic cell division region. Bar = 10 μm. **(I)** Ratio of seedlings showing ectopic cell division in pericycle and endodermis cells (*n* = 18 seedlings each). Data are significantly different from the PIN1 value at ^∗^*P* < 0.05 and ^∗∗^*P* < 0.0001 in *t*-test.

In vascular cells, WT PIN1, as expected, predominantly localized to the basal PM, whereas the basal localization of M3PIN1 was disturbed so as for the mutant PIN1 to be more localized to lateral sides than WT PIN1 (**Figure 1A**). Moreover, M3PIN1 tended to be more internalized than WT PIN1. Statistical analysis obviously revealed the defects of M3PIN1 and 3m1PIN1 in basal localization by showing considerably decreased ratio (~40%) of apical/basal to lateral localization (**Figures 1B,C**). In endodermal cells, while WT PIN1 localized predominantly in basal and inner lateral sides, mutation of M3 or 3m1 disturbed this polarity by delivering these mutant PIN1 proteins to the outer lateral PM (**Figures 1D–F**). These results indicate that the proper subcellular PIN1 polarity requires the M3 phosphorylation residues.

When roots were treated with the recycling inhibitor BFA, both WT PIN1 and M3PIN1 formed internal BFA compartments (**Figure 1G**), suggesting that, though M3PIN1 tends to internalize, some M3PIN1 proteins still are able to target to the PM and properly recycle like WT PIN1. This result in root meristematic vasculature cells is consistent with our previous observation that M3PIN1 and M3PIN7 are partially internalized and accumulated in BFA compartments in the root hair cell (Sasayama et al., 2013). However, M3PIN3 was almost completely internalized so as not to form BFA compartments in the root hair cell (Ganguly et al., 2012a). On the other hand, all 3m1 mutants of PIN1, PIN3, and PIN7 formed BFA compartments in the root hair cell as they were partially internalized (Ganguly et al., 2012a; Sasayama et al., 2013). These results, together with the developmental phenotypic effects as will be mentioned, suggest that the phosphorylatable residues in the M3 site function redundantly.

In a previous study, ectopic cell division was observed in the M3PIN3-expressing root pericycle (Ganguly et al., 2012a). M3PIN1 and 3m1PIN1 also caused considerable increase of ectopic cell division in the pericycle (**Figures 1H,I**). It is likely that the disruption of PIN subcellular polarity generally causes ectopic cell division probably by interfering local auxin concentration gradients.

### The M3 Phosphorylation Site Is Required for PIN1-Mediated Plant Development

The *pin1* mutant shows structural abnormalities in inflorescence axes, flowers, and leaves (Okada et al., 1991). To investigate the biological roles of M3 and 3m1 phosphorylation sites, the phenotypes of *pin1* complemented with M3PIN1 and 3m1PIN1 were analyzed.

**FIGURE 2:**
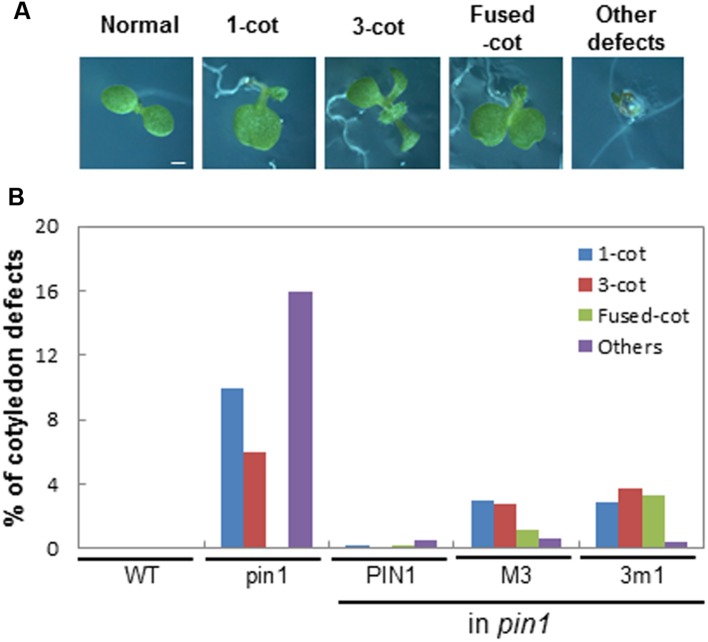
**Effect of M3 or 3m1 mutation of PIN1 on seedling development. (A)** Six-day-old seedlings showing cotyledon defects by PIN1 mutations. Bar = 0.5 mm for all. **(B)** Quantification of cotyledon defects shown in **(A)**. All the segregated F1 progeny seedlings of self-fertilized heterozygous *pin1* (±) mutant background were observed for *pin1* and *pin1* with PIN1 (*ProPIN1:PIN1:GFP*), M3PIN1 (M3, *ProPIN1:M3PIN1:GFP*), and 3m1PIN1 (3m1, *ProPIN1:3m1PIN1:GFP*). Total 403–485 seedlings from 7 to 8 independent lines per construct were observed. cot, cotyledon; WT, wild-type.

The loss of PIN1 caused aberrant cotyledon morphogenesis such as single, multiple, or fused cotyledons (**Figure 2A**), most likely by affecting auxin-gradient formation during embryogenesis. For the phenotypic analysis of cotyledons, the segregated F1 progeny seedlings of the self-fertilized heterozygous *pin1* mutant background were observed because homozygous M3PIN1-complemented *pin1* mutants barely produced seeds for next generation. While complementation with WT PIN1 greatly restored the normal cotyledon phenotype, complementation with M3PIN1 or 3m1PIN1 only partially rescued the *pin1* cotyledon defects (**Figure 2B**). Interestingly, the phenotype ratio of ‘fused cotyledon’ was rather increased by M3 or 3m1 mutation in comparison with the complete loss of PIN1 (*pin1*) (**Figures 2A,B**). Furthermore, some severe defects such as reduced or no cotyledons were observed by the complementation with both M3PIN1 and 3m1PIN1, indicating that these PIN1 mutants, by impairing its native polarity, lead to alteration of local auxin gradients and subsequent morphogenetic changes during embryogenesis.

**FIGURE 3:**
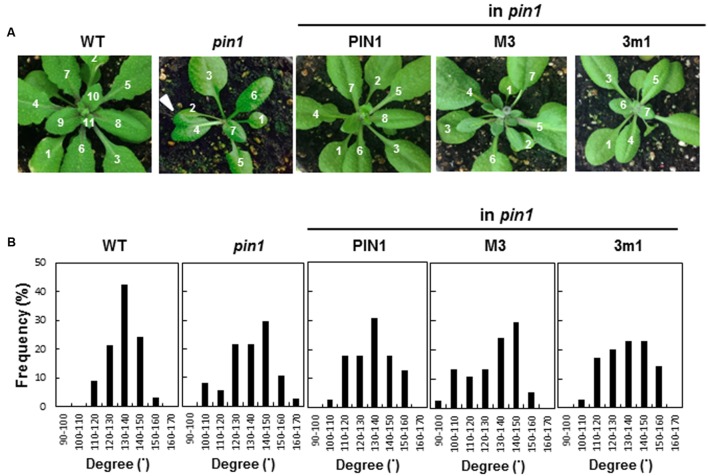
**Effect of M3 or 3m1 mutation of PIN1 on phyllotaxis of vegetative leaves. (A)** Rosette leaves of wild-type (WT), homozygous *pin1*, and homozygous *pin1* complemented with PIN1 (*ProPIN1:PIN1:GFP*), M3PIN1 (M3, *ProPIN1:M3PIN1:GFP*), and 3m1PIN1 (3m1, *ProPIN1:3m1PIN1:GFP*). Arrow head indicates fused leaf shown in *pin1* mutant. **(B)** Distribution of leaf-emerging angles in the plants described in **(A)** (*n* = 33–39 plants; in case of transgenics, from 5 to 7 independent lines for each construct).

Because PIN1 is associated with leaf initiation and phyllotaxis (Okada et al., 1991; Reinhardt et al., 2003; Prasad et al., 2011), we examined rosette leaf phyllotaxis of the complemented lines with WT PIN1, M3PIN1 or 3m1PIN1. WT plants showed a typical phyllotactic distribution pattern with the peak at 130–140° of leaf angle (**Figures 3A,B**). This distribution pattern was dissipated in the homozygous *pin1* mutant by considerably deviating its peak from the typical angle of 130–140°. Complementation of homozygous *pin1* with WT PIN1 restored the peak at 130–140°. However, M3PIN1- or 3m1PIN1-complementation failed to restore the WT phyllotactic peak, indicating that the M3 phosphorylation site is necessary for PIN1’s function in phyllotaxis.

**FIGURE 4:**
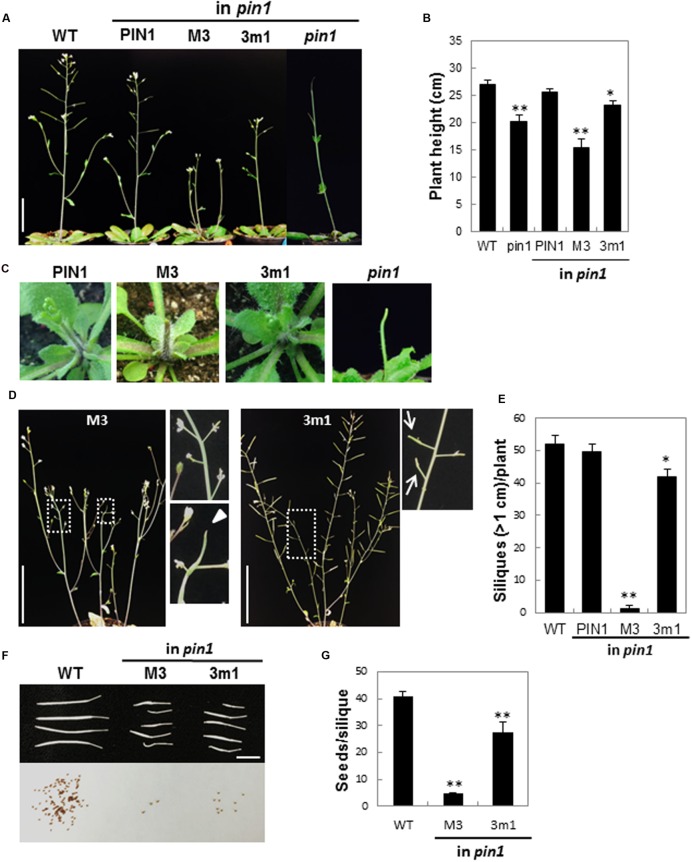
**Effect of M3 or 3m1 mutation of PIN1 on reproductive development. (A)** Inflorescence stems of WT, homozygous *pin1*, and homozygous *pin1* complemented with PIN1 (*ProPIN1:PIN1:GFP*), M3PIN1(M3, *ProPIN1:M3PIN1:GFP*), and 3m1PIN1 (3m1, *ProPIN1:3m1PIN1:GFP*). Bar = 5 cm. **(B)** Average height of the plants in **(A)**. Data represent means ± SE (*n* = 7–10). Differences are significant from WT value at ^∗^*P* < 0.05 and ^∗∗^*P* < 0.005. **(C)** Early developing inflorescence stems. **(D)** Defects in reproductive organs by M3 or 3m1 mutation. Arrowhead indicates the defect in the stem tip. Arrows indicate defects in siliques. **(E)** Number of silique over 1-cm long. Data represent means ± SE (*n* = 5–8 plants). Differences are significant from WT value at ^∗^*P* < 0.05 and ^∗∗^*P* < 0.005 in *t*-test. **(F)** Siliques and seeds from WT and strong phenotypic siliques of M3 and 3m1 plants. Seeds were harvested from the siliques shown in the upper photograph. Bar = 5 mm. **(G)** Number of seeds per silique. Data represent means ± SE (*n* = 8–20 siliques). Differences are significant from WT value at ^∗∗^*P* < 0.005 in *t*-test.

The homozygous *pin1* mutant forms a pin-like inflorescence stem lacking lateral shoot organs (Okada et al., 1991; **Figures 4A,C**). M3PIN1-complemented plants grew a pin-like primary inflorescence stem that, however, was soon aborted (**Figure 4C**). This early abortion of the primary stem by M3PIN1 led to growth of secondary inflorescence stems (**Figure 4A**). In contrast, 3m1PIN1-complemented plants kept growing the primary stem, although the size was smaller than that of WT plants (**Figure 4B**). The M3PIN1 plants, though growing secondary stems, eventually produced aborted shoot apexes in these secondary stems (**Figure 4D**). The homozygous *pin1* mutant did not abort the primary stem early but maintained its growth for a while (Okada et al., 1991; **Figure 4A**).

In addition to their effects on inflorescence stem development, M3 or 3m1 mutation also affected silique development so as to produce aberrant or no silique (**Figures 4D–F**). The WT plants generally produced over 1.5 cm siliques. However, the number of siliques over 1 cm was decreased considerably by 3m1 and greatly by M3 mutation (**Figure 4E**). Consistently, the seed number per silique also decreased by these mutations to 11% (M3) and 67% (3m1) of the WT level (40.9 seeds/silique) (**Figures 4F,G**).

These results together suggest more than that the M3 phosphorylation site is necessary for normal biological functions of PIN1. M3PIN1- and 3m1PIN1-complemented transformants revealed new phenotypes that are not shown in the *pin1* mutant, such as generation of fused cotyledons and early abortion of the primary inflorescence stem. As we described earlier for the root tissues (**Figure 1**), the partial loss of polarity or altered intracellular trafficking of M3PIN1 and 3m1PIN1 could cause spatial changes of auxin gradients, which subsequently leads to developmental defects.

### The M3 Phosphorylation Site Is Necessary for Gravity-Induced PIN7 Relocalization and Root Gravitropism

In the root columella cell, upon changes of gravity vector, PIN3 and PIN7 relocalize so as to redistribute auxin for gravitropic bending of the root (Friml et al., 2002b; Kleine-Vehn et al., 2010). Under a constant gravity vector, these PINs symmetrically distribute in the columella cell PM. However, changes of the gravity vector cause endocytosis of those PINs and selective recycling of them toward the PM side facing gravity direction (bottom), resulting in higher auxin flow and inhibition of cell growth at the bottom side of the root.

**FIGURE 5:**
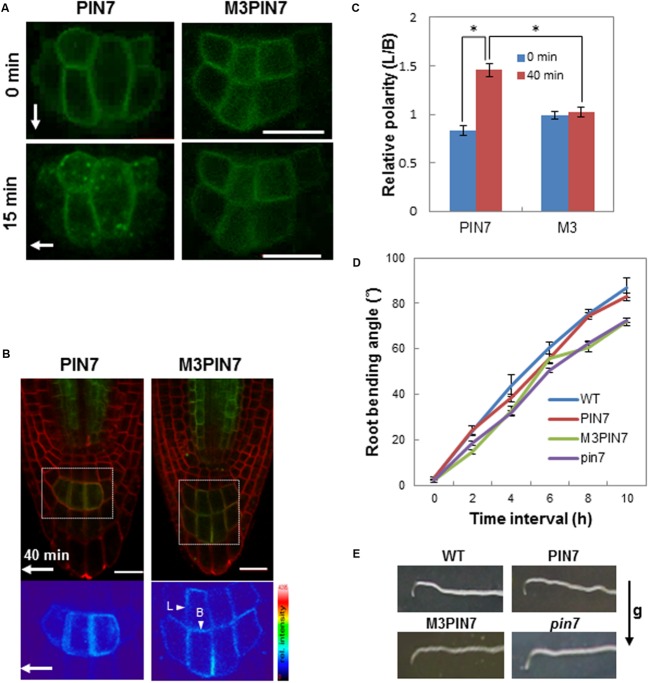
**Effect of M3 mutation on PIN7 trafficking and root gravitropism. (A)** Representative confocal fluorescence images of PIN7 (*ProPIN7:PIN7:GFP*) and M3PIN7 (M3, *ProPIN7:M3PIN7:GFP*) in root columella cells. Images were taken before (0 min) or after 90°-gravity stimulation for 15 min. Arrows indicate the gravity direction Bar = 20 μm. **(B)** Fluorescence images of PIN7 and M3PIN7 in root columella regions after 40-min gravity stimulation. Arrow indicates the gravity direction. Red signals were from FM4-64 staining. Bar = 20 μm. **(C)** The lateral/basal (L/B as indicated in **B**) ratio of PIN7 or M3PIN7 fluorescence intensity in columella cells at 0 min and after 40 min gravity stimulation. Data represent means ± SE (*n* = 12 cells from 6 roots for each construct). Asterisks indicate that differences are significant (*P* < 0.0001) in *t*-test. The difference of M3 values between 0 and 40 min was not significant (*P* = 0.631). **(D)** Kinetics of root gravitropism of WT, homozygous *pin7*, complemented with PIN7 (PIN7) and M3 (M3PIN7), and homozygous *pin7*. Data represent means ± SE (*n* = 29–40 roots). **(E)** Representative root images of the plants described in **(D)** after a 10-h gravity stimulus. The arrow indicates the gravity (g) direction.

Here, we tested whether the M3 phosphorylation site is implicated in PIN7’s function during root gravitropism. Similarly as in the PIN1 study, WT or M3-mutated PIN7 with the GFP tag under the *PIN7* promoter was complemented into the homozygous *pin7* mutant. We first observed the intracellular trafficking behavior of WT PIN7 and M3PIN7 upon gravity vector change. WT PIN7 accumulated into endocytotic vesicles within 15 min after gravity stimulus of the root whereas M3PIN7 did not show obvious accumulation in the endocytotic vesicle upon the gravity stimulus (**Figure 5A**). Before gravity stimulus, WT PIN7 and M3PIN7 were symmetrically localized at the columella cell PM (**Figure 5A**). After a 40-min gravity stimulus, WT PIN7 asymmetrically redistributed to the PM side facing to the new gravity direction (**Figures 5B,C**). However, M3PIN7 did not change its symmetrical distribution after the same gravity stimulus (**Figures 5B,C**). For a statistical analysis, we took the ratio of PIN7 signal intensity between lateral (L) and basal (B) sides of the columella cell (L/B, **Figures 5B,C**) as a polarity parameter. While WT PIN7 significantly increased its lateral localization from 0.83 to 1.46 of L/B after the 40-min gravity stimulus, M3PIN7 maintained almost the same ratio of L/B (~1.0) regardless of the gravity stimulus, suggesting that phosphorylation of the M3 site is essential for gravity-stimulated transcytosis and relocalization of PIN7 in the root columella cell.

Next we examined whether the defects of M3PIN7 in subcellular relocalization affect the gravitropic response of the M3PIN7 plant root. The *pin7* mutant plants exhibited root gravitropic defects as reported previously (Kleine-Vehn et al., 2010; **Figures 5D,E**). While complementation with WT PIN7 rescued root gravitropism of *pin7*, complementation with M3PIN7 failed to rescue (**Figures 5D,E**). In the gravitropic kinetics analysis of the root, the M3PIN7-complemented mutant showed relatively decreased gravitropic responses compared to WT and WT PIN7-complemented plants (**Figures 5D,E**). These results suggest that the M3 phosphorylation residues are necessary for PIN7-mediated root gravitropism most likely by modulating the transcytotic relocalization of PIN7 after gravity stimuli.

### M3 Mutation Affects PIN2 Protein Levels in the Root Epidermal Cells

**FIGURE 6:**
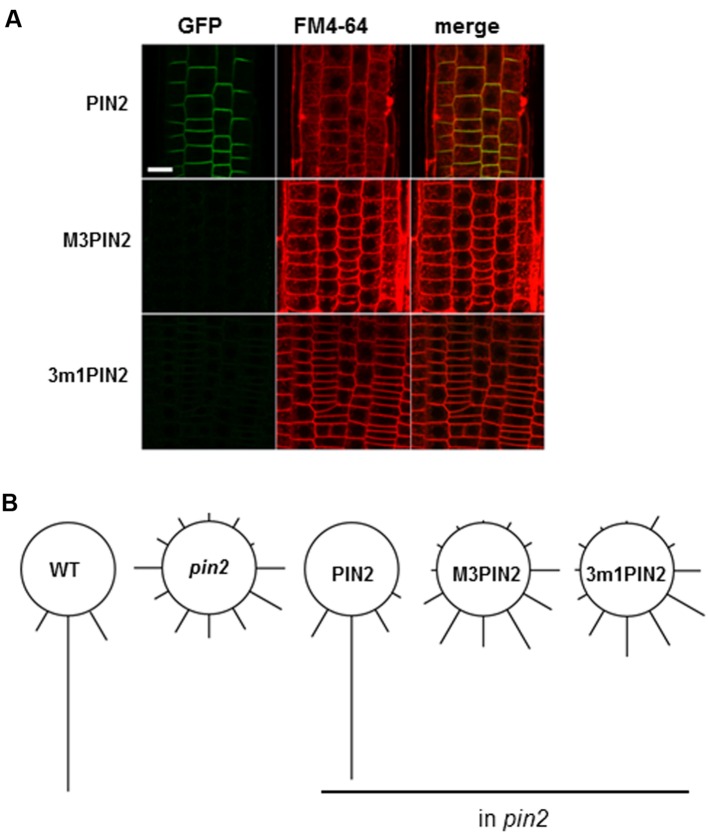
**The effect of M3 or 3m1 mutation on PIN2 trafficking and PIN2-mediated root gravitropism. (A)** Representative confocal images of the root epidermal cells of homozygous *pin2* complemented with PIN2 (*ProPIN2:PIN2:GFP*), M3PIN2 (*ProPIN2:M3PIN2:GFP*), and 3m1PIN2 (*ProPIN2:PIN2:GFP*). Bar = 5 μm. **(B)** Relative degrees of root gravitropism of wild-type (WT), homozygous *pin2*, and homozygous *pin2* complemented with PIN2, M3PIN2, and 3m1PIN2 after 24-h gravitropic stimulation (*n* = 44–135 seedlings).

As conducted for PIN1 and PIN7, we analyzed the homozygous *pin2* mutant plants complemented with WT PIN2, M3PIN2, or 3m1PIN2 with the GFP tag under the PIN2 promoter. WT PIN2 polarly localized to the upper side of the root epidermal cell PM as previously reported (Müller et al., 1998; **Figure 6A**). However, M3PIN2 showed almost no expression and 3m1PIN2 showed weak expression (**Figure 6A**), which is consistent with our previous result showing that M3PIN2 and 3m1PIN2, when expressed under a root hair-specific promoter (*ProE7*), did not express in the root hair cell (Sasayama et al., 2013). Accordingly to this results, the *pin2* mutant plant complemented with M3PIN2 or 3m1PIN2 failed to restore root gravitropism whereas the complementation with WT PIN2 restored it (**Figure 6B**). Intriguingly, the treatment with wortmannin, an inhibitor for vacuolar trafficking, was reported to restore both M3- and 3m1PIN2 protein signals in the cytoplasm but not in the PM of the root hair cell, suggesting that the M3 phosphorylation site is implicated in regulation of vacuolar targeting of PIN2 (Sasayama et al., 2013). These results suggest that a similar phosphorylation motif of PIN-HL can play different roles depending on PIN species.

Although the phosphorylatable Ser/Thr residues in long PINs are mostly conserved, the flanking residues vary particularly in PIN2 (Supplementary Figure S1). For the M3 region around the first two Ser residues, while TVRK(/R)SNAS is the consensus sequence in other long PINs, VVK(/R)RSXAS is the consensus of PIN2 orthologs. For the third Ser, PIN2 includes NKS rather than RRS. Finally for the region around forth Thr and fifth Ser, the PIN2 consensus is TPRAS whereas other long PINs have TPRPS. Although it has remained to be characterized, these syntactic variations around the M3 phosphorylatable residues might lead PIN2 to have different trafficking behavior most likely by different reader proteins against these different syntaxes around the phosphorylation residues.

### The M3 Site Is Required for PID-Mediated Phosphorylation of PIN1

**FIGURE 7:**
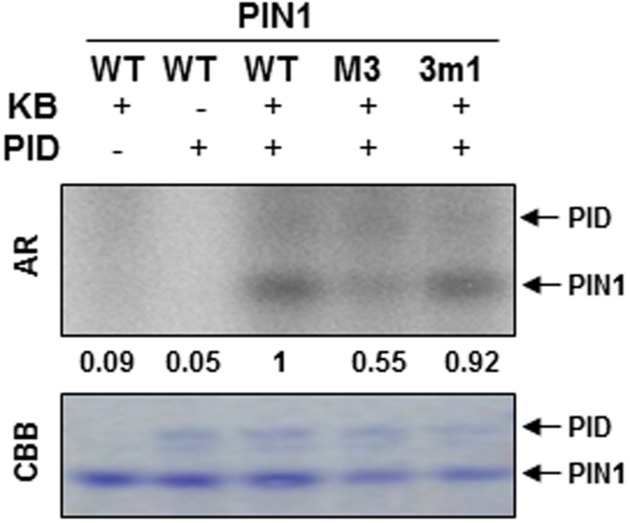
***In vitro* phosphorylation assay of the hydrophilic loop (HL) of PIN1.** Autoradiogram (AR) of PIN1-HL phosphorylation assay. Wild-type (WT), M3, and 3m1 PIN1-HL proteins were analyzed for phosphorylation by PID kinase using [γ-^32^P] ATP as phosphate source. KB, kinase buffer; CBB, Coomassie Brilliant Blue-stained gel. The numbers below AR indicate relative phosphorylation intensities of PIN1 that were normalized by the PIN1 band intensities in CBB.

In order to know whether the 5 Ser/Thr residues of the M3 site are involved in phosphorylation of PIN1, we performed *in vitro* phosphorylation assays with heterologously expressed PID kinase and M3- or 3m1-mutated PIN1-HL domain as kinase substrate and [γ-^32^P] ATP as a phosphate source. PID showed autophosphorylation (**Figure 7**). Compared with the WT PIN1-HL phosphorylation level, PID-mediated phosphorylation of M3-mutated PIN1-HL was considerably decreased (**Figure 7**). Conversely, 3m1 mutation marginally decreased the phosphorylation level (**Figure 7**). In our previous study with PIN3, M3 mutation showed a greater effect than 3m1 mutation on PID-mediated phosphorylation of PIN3-HL (Ganguly et al., 2012a). These results suggest that 5 Ser/Thr residues in the M3 site of long PINs are redundant targets of PID. Because the M3 mutation could not completely inhibit PID-mediated phosphorylation of the long PINs, PID is likely to phosphorylate other residues of the HL domain. The Ser residues of 3 repeats of TPRXS have been reported as the PID’s phosphorylation targets outside the M3 site (Dhonukshe et al., 2010; Huang et al., 2010). Therefore, this study consistently shows that the M3 site of long PIN-HL is a partial target of PID.

Although the native function of M3 phosphorylation site was studied with PIN3 (Ganguly et al., 2012a) and the role of M3 site of PIN1, 2, and 7 in subcellular trafficking was shown in the root hair model system (Sasayama et al., 2013), the native function of M3 site of PIN1, 2, and 7 in their own expression domains had not been characterized. Our current study, by analyzing the native biological roles of M3 phosphorylation site of PIN1, 2, and 7, demonstrates that the M3 phosphorylation site is generally required for proper trafficking and intracellular polarity, native biological functions, and PID-mediated phosphorylation of long PINs.

## Author Contributions

H-TC designed the project. DK performed most of the experiments for plant analyses, microscopy, and biochemistry. DS made the transgenic constructs. All authors contributed to the interpretation of results and writing of the manuscript. All authors approve the manuscript publication.

## Conflict of Interest Statement

The authors declare that the research was conducted in the absence of any commercial or financial relationships that could be construed as a potential conflict of interest.

## Footnotes

1 https://imagej.nih.gov/ij/
