# Supplementary material for: The M3 Phosphorylation Site Is Required for Trafficking and Biological Roles of PIN-FORMED1, 2, and 7 in Arabidopsis
Source: Front Plant Sci. 2016 Sep 28;7:1479. doi: 10.3389/fpls.2016.01479 (PMC5039202; doi:10.3389/fpls.2016.01479)
Supplement: Supplementary file 1 [file Table_1.PDF]

Table S1. Primer list

Subject	Template	Primer Name	Sequence (5' to 3')
PIN1 promoter	Genomic DNA	PIN1PR-Hd-F	GTT TAA GCT TCC ATA ACC ATA AGT CAA GCC GTG C
		PIN1PR-Sl-R	GTT TGT CGA CCT TTT GTT CGC CGG AGA AGA GAG
PIN2 promoter	Genomic DNA	pPN2-PmeI-F	CGACGTTAAACTGCAAGGATATCATTACCAGTACCG
		pPN2-SalI-R	CTGGTCGACTTTGATTTACTTTTTCCGGCGAGAG
PIN7 promoter	Genomic DNA	PIN7PR-Hd-F1	GTT TAA GCT TCT CTC TTT CTT CAG TGA TG
		PIN7PR-Sl-R	TTA GTC GAC ATT GTT GTT CGC CGG AGT GG
PIN1-HL, M3PIN1-HL, 3m1PIN1-HL	Complementary DNA	PIN1-CL-Bm-F	TATAGGATCCGACACAGCAGGATCTATTG
		PIN1-CL-Sl-R	TATAGTCGACTCACACTTGTTGGTGGCATC
