# Supplementary figures and images for: The M3 Phosphorylation Site Is Required for Trafficking and Biological Roles of PIN-FORMED1, 2, and 7 in Arabidopsis
Source: Front Plant Sci. 2016 Sep 28;7:1479. doi: 10.3389/fpls.2016.01479 (PMC5039202; doi:10.3389/fpls.2016.01479)

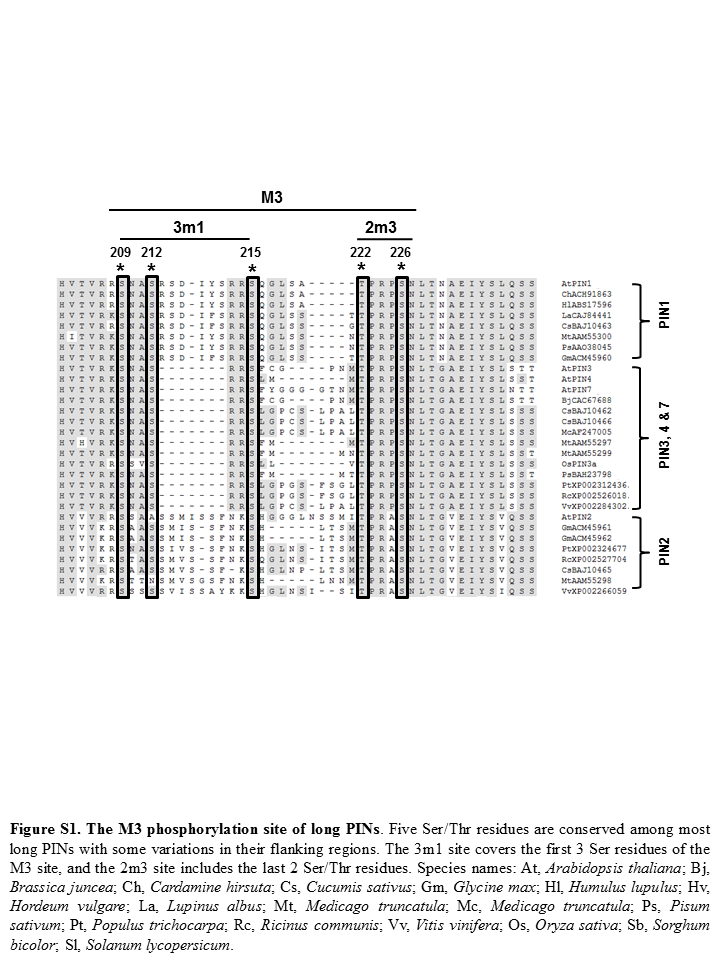

Supplement: Supplementary file 2 [file Image_1.TIF]
